# Monitoring saltwater intrusion in Rupert Bay, Québec, Canada, after the partial diversion of a major tributary

**DOI:** 10.1007/s10661-017-6388-2

**Published:** 2017-12-21

**Authors:** Vincent Métivier, Bernard Massicotte, Alain Tremblay, Pierre Dupuis

**Affiliations:** 1 Energy Unit, WSP Canada, Québec City, QC Canada; 2 Environmental Unit, WSP Canada, Québec City, QC Canada; 3 Hydro-Québec, Montréal, QC Canada; 4 Energy Unit, WSP Canada, Laval, QC Canada

**Keywords:** Saltwater intrusion, Monitoring, River diversion, Ice cover

## Abstract

As part of a large hydroelectric project in northern Québec (Canada), a portion of the flow of the Rupert River was diverted toward the existing La Grande hydroelectric complex. As a result of the partial diversion, the discharge of the Rupert River at its mouth is reduced by an average of 50% annually. This corresponds to an 18% decrease in the total freshwater inflow into the bay and, thus, to a shift of the upstream limit of the saltwater intrusion in Rupert Bay. Changes in saltwater intrusion had been predicted numerically as part of the project’s environmental impact assessment (EIA). In the project’s conditions of authorization, monitoring the hydraulic conditions and the extent of saltwater intrusion in the Rupert Bay was required by government authorities. The objective of this paper is to present the results of this environmental monitoring and, more specifically, to validate the modifications predicted in the EIA in terms of both saltwater intrusion limit and hydraulic conditions in the Rupert Bay. Results obtained during 2 years of monitoring are within the predicted trends and order of magnitude of changes anticipated in the EIA. The results, thus, confirm that the shift of the upstream limit of the saltwater front along the channels of the bay was conservatively predicted by numerical modeling.

## Introduction

Lakes, rivers, and estuaries as well as wetlands and forests are part of the Earth’s freshwater cycle. Generally, in Quebec, the largest river runoffs take place from April to June as snow melts in spring, the smallest runoffs occurring during winter. It is well-known that the creation of reservoir either for water management (irrigation, flood control, etc.) or for energy generation (reservoir, pump station) can have a significant effect on the river hydrological cycle by changing this general pattern (Prinsenberg, 1994, Rosenberg et al., 2000). Canadian winters correspond to high electricity demand that results in the release of a large amount of freshwater to the estuary that may have implications for plume surface area as well as estuarine dynamics (Dunbar, 1982; Messier and Anctil, 1996, Hayeur, 2001).

The Connecticut River (Garvine, 1974, 1975, 1977), La Grande Rivière, Great Whale River (e.g., Ingram and Larouche, 1987a, Prinsenberg 1994), and Fraser River (Cordes et al. 1980) are some examples of studied river plumes. In river systems where the ratio of mean freshwater discharge velocity to tidal current at the river’s mouth is large, extensive plumes are formed at all times of the year (Prinsenberg 1982, 1994). Plumes are also present during winter and early spring, when runoff values are at their smallest levels (Ingram, 1981). For ice-free periods, Garvine (1975) showed that the surface area of the Connecticut River plume varied in an approximately linear manner with volume of discharged freshwater. Similarly, with 42 river discharge data from 1966 to 1994, Déry et al. (2005) suggested a relationship between freshwater discharge and salinity. They suggested that a gradual salinization of the upper ocean during summer over the period of 1966–1994 on the inner Newfoundland Shelf is in accord with a decadal trend of a diminishing intensity in the continental meltwater pulses. Plumes are also affected by tidal amplitude and costal circulation as observed by Cordes et al. (1980) in the Fraser estuary and by Ingram and Larouche (1987a) in Hudson Bay. Wind is another parameter known to influence dynamics and flushing rates of shallow estuaries as reported by Geyer (1997).

Knowledge of under-ice plumes is fairly recent. Most of the studies were undertaken on the east coast of the James and Hudson Bays, motivated by the development of hydroelectric potential of northern Quebec. In that regard, Freeman (1982), Ingram (1981), and Ingram and Larouche (1987a) on Great Whale River as well as Messier et al. (1986, 1989) and Ingram and Larouche (1987b) on La Grande Rivière have shown that there is a marked increase in plume area for a given freshwater discharge under sea ice compared to open-water conditions.

The main justifications invoked to explain the vertical and horizontal extents of the winter plumes are the suppression of wind mixing and attenuation of tidal mixing (Ingram 1981, 1983; Freeman et al. 1982, Lepage and Ingram, 1991) reducing the overall energy available for mixing, which results in a much more stable interface between fresh and ambient water masses.

As part of the Eastmain-1-A, Sarcelle, and Rupert diversion project that was commissioned in 2009 by Hydro-Québec, a portion of the Rupert River water flow was diverted to the Eastmain 1 reservoir, through a series of diversion canals and tunnels. The discharge of the Rupert River at its mouth into Rupert Bay is thus reduced to an average of 50% annually. The potential impacts of this hydrological change have been the subject of specific studies conducted as part of the Eastmain-1-A-Sarcelle-Rupert project’s environmental impact assessment (EIA) (Hydro-Québec, 2004). As part of this study, 2D hydraulic modeling was conducted to predict the effect of the diversion on the intrusion of saltwater within Rupert Bay. The hydrodynamic model Mike 21 HD™, from the Danish Hydraulic Institute (DHI), was used to describe the hydrodynamic regime of the Rupert Bay (Dupuis, 2004). The numerical model predicted a displacement of the maximum intrusion of the saline front 5 km upstream within Rupert Bay and no noticeable variation in water levels in the bay (Dupuis, 2004; Hydro-Québec, 2004). Furthermore, a slight shift of the freshwater flow corridors from the Nottaway, Rupert, and Broadback Rivers in Rupert Bay toward the northeast was predicted (Hydro-Québec, 2004). Predictions provided insights into physical processes and assessed impacts of diversion of a major tributary of the Rupert Bay. Few years after the diversion of the Rupert River, one could ask the following:

1. How does the limit of saline intrusion differ from the post-diversion baseline, in both ice-free and ice cover conditions?
2. How do the changes predicted in the EIA compare to post-diversion measurements?

Monitoring of saltwater intrusion in post-diversion conditions was undertaken in 2010 and 2013 to answer these questions. The objective of this paper is to present the results of this environmental follow-up, more specifically, to validate the modifications predicted in the EIA in terms of both saltwater intrusion limit and hydraulic conditions (water levels) in the Rupert Bay in both ice-free and ice cover conditions.

With the increasing pressure exerted on freshwater resources, it is more than ever important to improve the methodology and the empirical knowledge of the effects of river diversions. This paper will hopefully benefit researchers and environmental managers interested in the prediction and validation of the effects of river diversions on oceanographic processes.

### Study area

The Rupert Bay is in the boreal ecoregion of Québec, Canada, about 800 km north of Montréal. The watershed is dominated by coniferous forest and shallow podzolic and peat soils developed over igneous bedrock and quaternary sediments. Aquatic systems are described as oligotrophic—characterized by a low water nutrient concentration and a high oxygen content. The hydrology of the Rupert Bay watershed (139,465 km^2^ in natural conditions (Hydro-Québec, 2004)) reflects the regional climate; runoff is strongly seasonal, with high flows in the spring (peaking in May or June) and low flows in late winter.

**Fig. 1:**
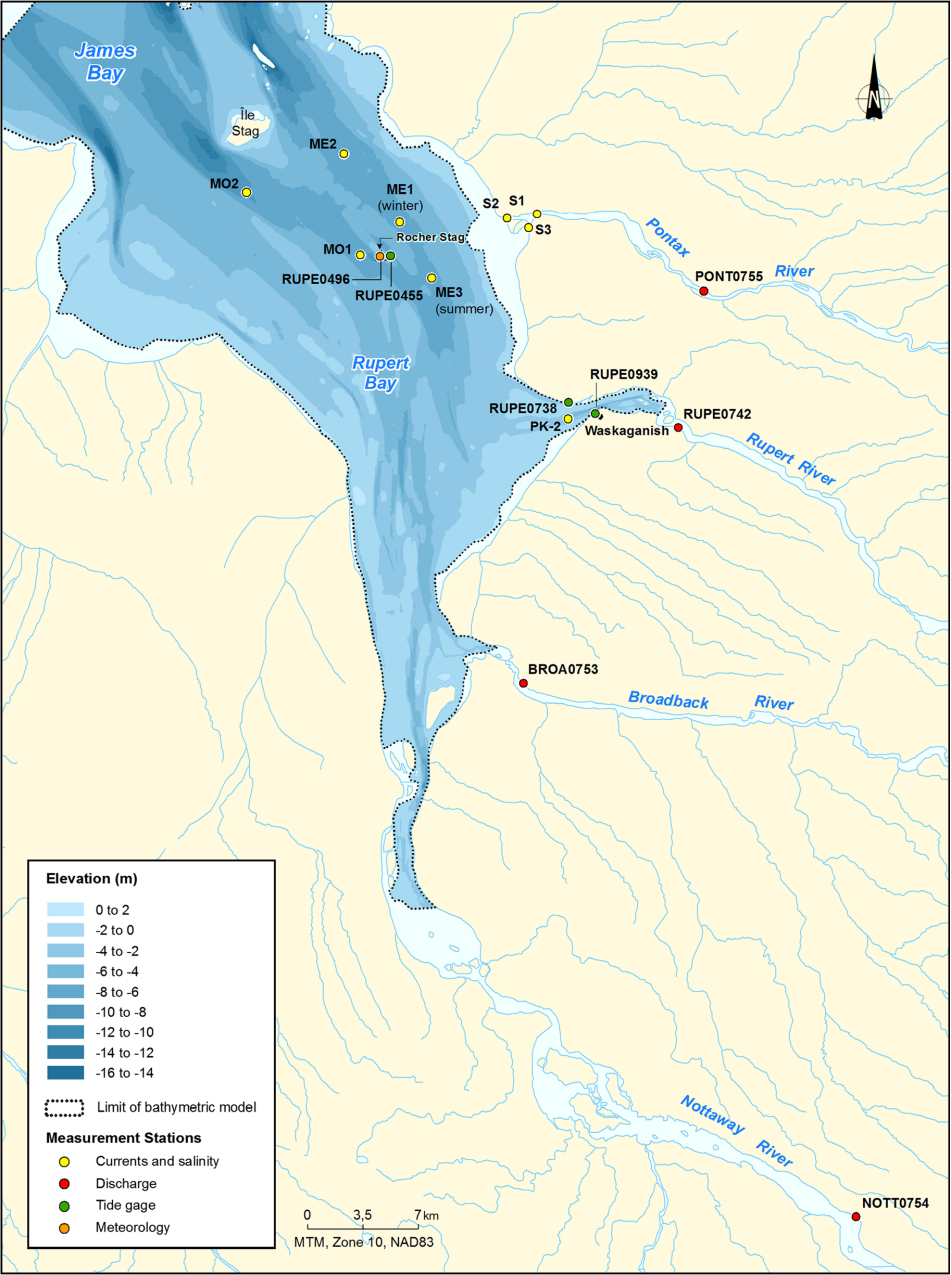
Location of data-logging stations

**Fig. 2:**
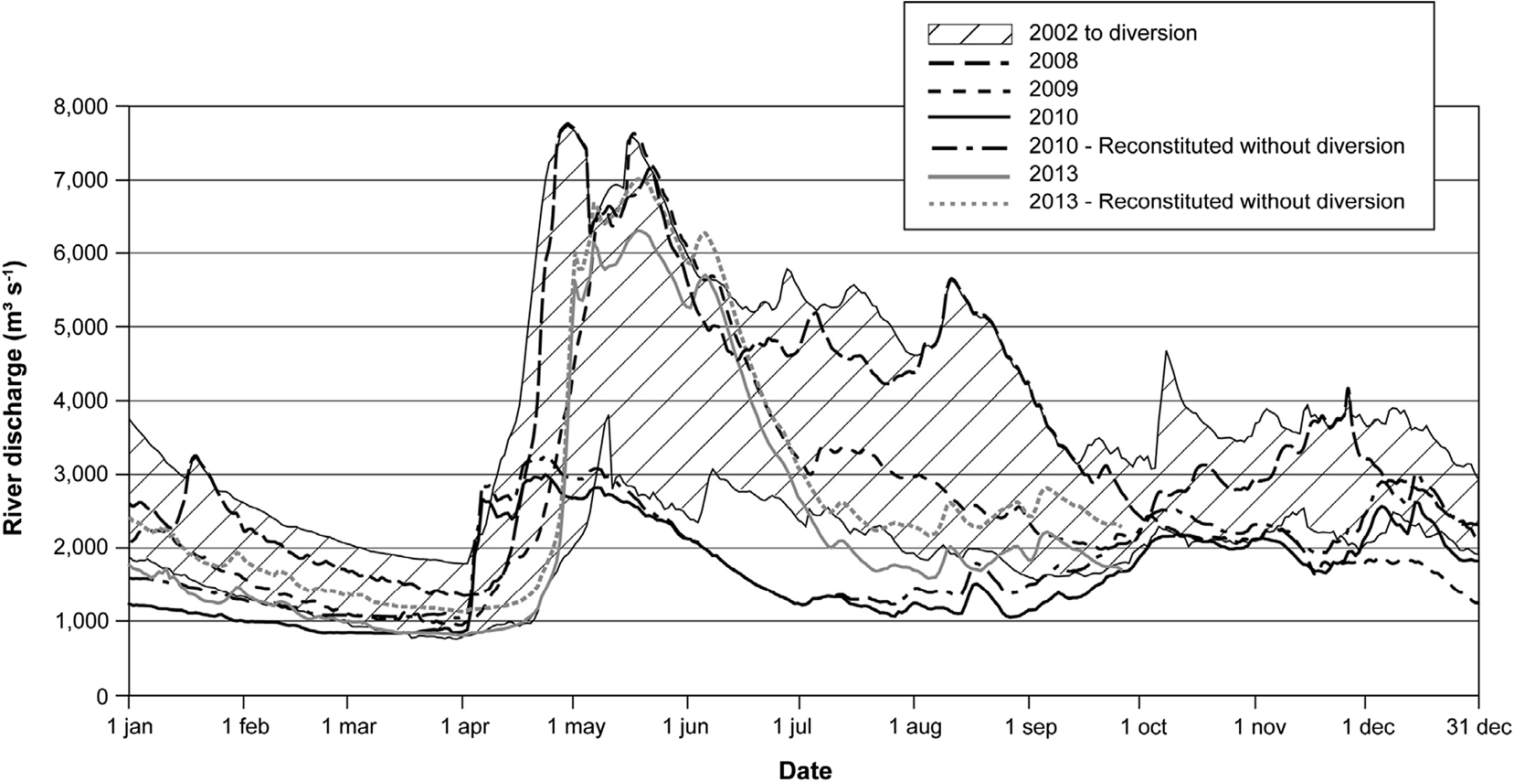
Daily total and reconstituted discharges in Rupert Bay (daily total discharge from Nottaway, Broadback, Rupert, and Pontax rivers) during 2008, 2009, 2010, and 2013 without diversion

Rupert Bay is a shallow (3–5 m), turbid river estuary located off the southeast of James Bay (Fig. 1). It can be divided into three zones: freshwater, brackish water, and saltwater zones. In the freshwater zone, it received a mean annual freshwater inflow varying from 1000 to more than 7000 m^3^ s^−1^ from four major tributaries, the Nottaway, Broadback, Rupert, and Pontax Rivers (Figs. 1 and 2). The decrease in the Rupert’s discharge, following the diversion, corresponds to an 18% decrease in the total freshwater inflow to the bay. To reduce the impact of the project on the Rupert and Lemare Rivers, eight weirs were built on the Rupert River to maintain the water level for different uses of the river such as navigation and fish spawning areas, and an ecological instream flow that reproduces the natural hydrological cycle on both rivers is maintained.

Characterization of freshwater inflows is required as it plays a significant role in the saltwater intrusion pattern found over Rupert Bay. The combination of shallow depths, bay dimension, and large quantities of freshwater inflows creates zones of fresh and brackish waters within the bay. The saltwater boundary moves upstream and downstream under the influence of tidal forcing and freshwater inflow that comes mainly from the Nottaway, Broadback, Rupert, and Pontax Rivers.

Prior to the 2009 diversion, the smallest freshwater inflow occurred late in winter and was in the order of 1000 to 2000 m^3^ s^−1^. In summer, smallest inflows ranged between 2000 and 5000 m^3^ s^−1^ (Fig. 2). For the same periods, post-diversion inflows vary in the order of 900 to 1500 m^3^ s^−1^ in winter and fluctuate between 1000 and 2000 m^3^ s^−1^ in summer. In the period of spring freshet, total river discharges ranged from 2700 to 7500 m^3^ s^−1^ (pre- and post-diversion 2008–2013).

In 2013, freshwater discharge from the four main tributaries ranged between 786 and 6272 m^3^ s^−1^ (Consortium Waska-Genivar, 2014). Spring freshet spanned the months of May and June. Between February 10th and April 3rd, the mean total freshwater inflow was 933 m^3^ s^−1^. It ramped up to 1746 m^3^ s^−1^ between July 22nd and August 26th. Despite the diversion, freshwater inflows in Rupert Bay were larger in 2013 than in 2010, especially during the freshet and in summer. With respect to the winter period, a comparison of freshwater inflows shows that the total discharge evaluated in 2013 was slightly larger than in 2010. This analysis shows that 2010 was not a typical post-diversion year, the incoming flow being much smaller in 2010 than those evaluated for the previous 2 years.

In post-diversion conditions, freshwater inflows are found to be less for the periods of February–March and July–August than those of pre-diversion conditions. From January 1st to mid-March 2010 as well as June to September 2010, the total inflow was below the lower envelope curve of inflow for the period of pre-diversion. Furthermore, reconstituted freshwater inflows for conditions without diversion show that for 2010, flows were also below this curve for the months of January, June, July, and August. Low freshwater inflow in Rupert Bay is related to a low runoff for this year. Owing to the relatively large volume of the bay, these smaller freshwater inputs had no discernible effect on the water level as will be shown hereafter.

The mean annual air temperature is in the order of 0.5 to 3 °C and varies between − 33 °C in the winter and 31 °C in the summer. Atmospheric pressure varies generally between 97.9 to 103.4 kPa with westerly winds dominating with speeds from 5 to 40 km/h, and wind peaks ranged from 60 to 80 km/h but can go up to 100 km/h for short periods of time (Consortium Waska-Genivar, 2014).

Semidiurnal tides play a significant role in the water level variations of the bay (Consortium Waska-Genivar, 2014). In open water, water levels generally vary between − 1 and 1 m (MSL), while under an ice cover, they generally range between − 0.5 and 0.5 m (MSL) (Environnement Illimité INC, 2011, Consortium Waska-Genivar, 2014). Water level variations in Rupert Bay are also influenced by weather conditions. For example, on August 22nd, 2003, water levels of up to 4 m (MSL) were observed when northwest winds exceeding 100 km/h and atmospheric pressure below 99 kPa were measured (Dupuis, 2004).

## Materials and methods

The effect of the Rupert diversion on the intrusion of saltwater was assessed as part of a larger program aiming to understand the environmental effects of the Rupert River diversion. Hydro-Québec has been operating weather, water level, and limnimetric stations in Rupert Bay (Fig. 1), for over 10 years, thus both before and after the diversion, which occurred in November 2009. The data records from these stations that were used in the present study are indicated in Table 1.

**Table 1:** Summary of the data used to compare the saltwater intrusion in Rupert Bay in pre- and post-diversion conditions

Data	Data logging frequency	Data averaging	Pre-diversion (natural conditions)	Post-diversion year 1	Post-diversion year 4
Air temperature	15 min	Hourly	Jan. 2008–Nov. 2009	Nov. 2009–Dec. 2010	Jan.–Oct. 2013
Atmospheric pressure	15 min	Hourly			
Wind velocity and direction	15 to 60 min	Hourly			
Freshwater discharge	Daily	None			
Water levels	15 to 60 min	Hourly			
Salinity	1 min for moorings; < 10 s for discrete measurements and manual measurements	No temporal averaging for moorings; water column averaging for discrete measurements	Mar. 2003	Feb.–Apr. 2010	Feb.–April 2013
Current velocity and direction	10 to 15 min	Hourly	Aug.–Sept. 2003	Aug.–Sept. 2010	Jul.–Aug. 2013

Salinity data, on the other hand, are not regularly collected in Rupert Bay. The salinity data used in this study stem from data collection programs, which were conducted prior to the diversion, in 2003, and after the diversion, in 2010 and 2013, as part of the present study.

For post-diversion conditions, a monitoring protocol was developed in 2007 and oceanographic moorings and field campaigns were conducted both in the winters and summers of 2010 and 2013. The data from these campaigns, unpublished yet, were collected specifically for the purpose of this study.^1^ The measurements were made during periods when saltwater intrusion is expected to be the greatest, with the goal of capturing the maximum intrusion of the front. For both post-diversion years, measurements were made at the end of winter and summer, the two periods of the year when freshwater discharge into the bay is the smallest. Also, the four salinity and current measurement campaigns were carried out during a spring tide, when the inflow of saltwater into the bay is, in theory, greater. The data collected in post-diversion conditions for the purpose of this study are detailed in the following sections.

### Rupert Bay

The measurements in Rupert Bay included continuous recordings at four mooring stations as well as discrete measurements along the main channels and the shores. Two mooring stations were installed along the west channel and two along the east channel (Fig. 1). Each mooring station included a subsurface and bottom CTD (conductivity, temperature, and depth) sonde (RBR Concerto) and an acoustic Doppler current profiler (ADCP Sentinel Workhorse 600 and 1200 kHz). The moorings were deployed for periods ranging from 35 to 49 days comprising two spring tides.

**Fig. 3:**
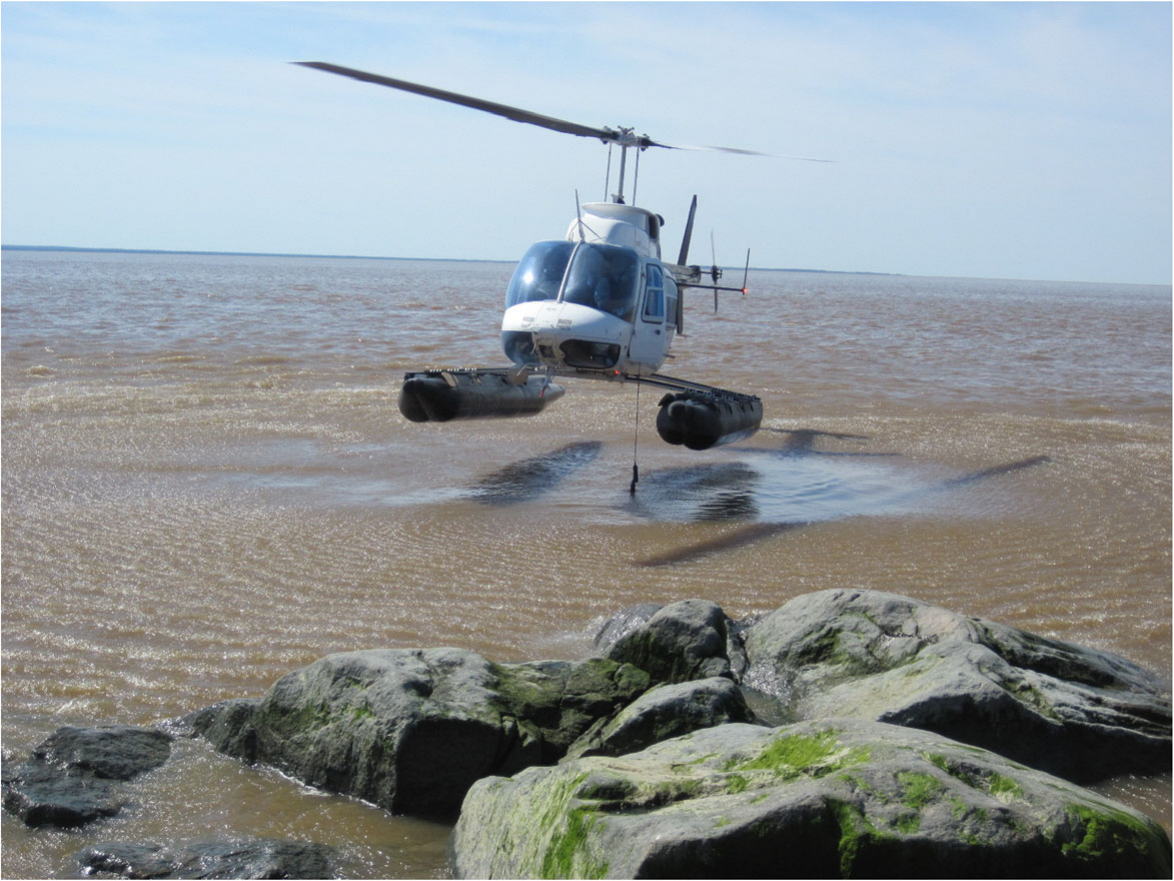
Locating the saltwater front near the shore

During each of the four post-diversion campaigns, two or three mobile teams simultaneously made discrete measures at specific stages of the tidal cycle to locate the maximum extension and retreat of the saline front. The measures consisted in CTD profiles using RBR XR-420 and RBR XR-620 loggers, additional salinity measurements (YSI 556 and YSI Professional Plus multisondes), and current velocities (Rio Grande Workhorse 600 and 1200 kHz ADCPs). The measures were made along the channels during each campaign and also along the shores during the summer campaigns. Table 2 shows the number of vertical salinity profiles conducted in pre- and post-diversion conditions. In order to follow the salinity front during the tide cycles over distances of kilometers, the teams used helicopters during winter and summer campaigns as well as boats during the summer campaign (Fig. 3).

**Table 2:** Number of vertical salinity profiles conducted to locate the saltwater front in Rupert Bay, in pre- and post-diversion conditions

Period	Pre-diversion	Post-diversion	
	2003	2010	2013
Winter (ice cover)	N/A	223	164
Summer (open water)	220	230	544

The bottom depth in the surveyed area was generally between 3 and 7 m. For the sake of locating the saltwater front, the water column was thus considered as well-mixed. In this study, the saltwater front is defined by a salinity of 0.5 averaged over the water column.

### Mouth of the Pontax River

The saltwater intrusion was also monitored at the mouth of the Pontax River, a tributary of Rupert Bay. One multiparameter sonde (InterOcean System S4) was moored upstream from the mouth and two more in the main channels downstream from the mouth (Fig. 1). The sondes were mounted on a rack and moored on the sea floor. The variables monitored were salinity and current velocity and direction.

### Weather, freshwater discharge, and tidal data

Data on air temperature, atmospheric pressure, and wind velocity and direction were obtained from a weather station located on Stag Rock (Fig. 1), operated by Hydro-Québec. Additional data on air temperature and atmospheric pressure from the nearby Waskaganish weather station were used where gaps existed in the Stag Rock data.

Freshwater discharge data were obtained from the gauging stations operated by Hydro-Québec in the main tributaries of Rupert Bay, i.e., the Nottaway, Broadback, Rupert, and Pontax Rivers, for the pre- and post-diversion periods.

Water level data for both periods were obtained from the Stag Rock station and from two stations located at the mouth and 2 km upstream from the mouth of the Rupert River.

### Data analysis

**Fig. 4:**
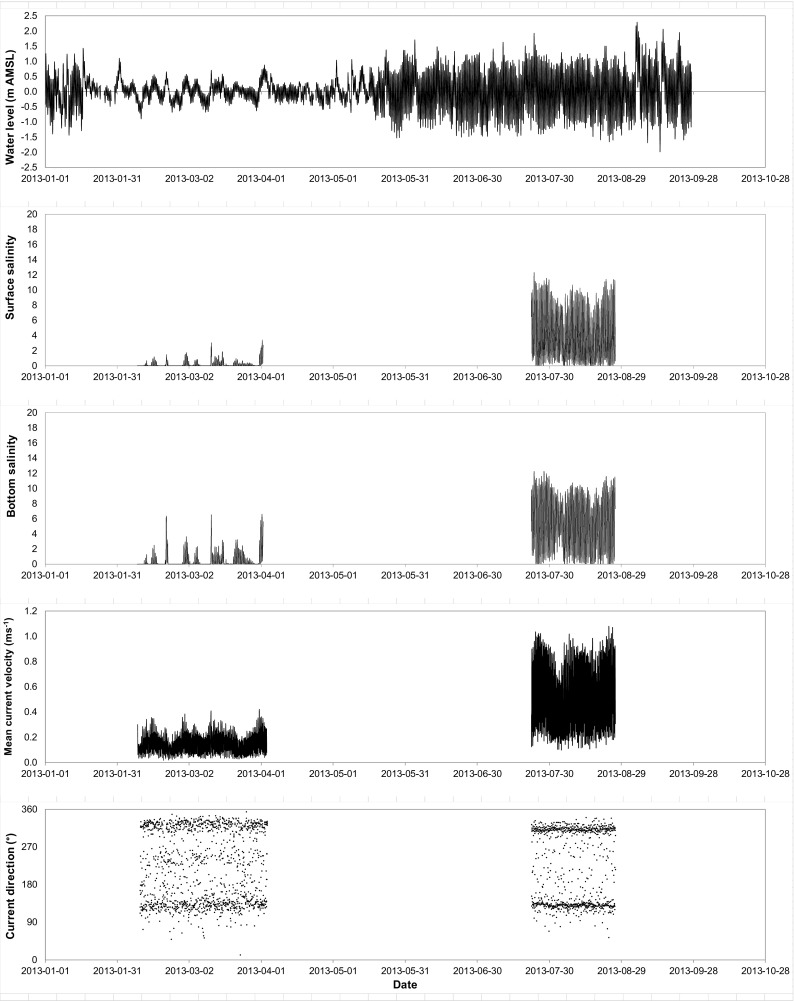
Time series of the water level at Stag Rock (RUPE0455), surface and bottom salinities, and current velocity and direction at station MO2

The time series of weather, river discharge, water level, and salinity data was examined for gaps and outliers. Figure 4 presents an example of water level, salinity, and current time series. Short periods of missing data (e.g., less than a tidal cycle or 5 h) were completed using linear interpolation. Longer data gaps were completed by transposition of the data recorded at a nearby station.

Water levels in the Rupert Bay were examined using a cumulative sum method (Dupuis 2010) to detect any vertical translation of the sensors. Interannual variations of the main tidal constituents were analyzed using tidal harmonic analysis. Sixty harmonic components of the tidal cycle were assessed using the Aquawave software (GENIVAR, 2011, implementation in C++ of Foreman development (1977)) for a time series of 35 days or more. To assess the effect of meteorological disturbances on water levels, a low-pass filter (A_24_A_24_A_25_, Godin 1972) was applied to the series in order to remove the tidal signal. Pre- and post-diversion conditions were compared using water level duration curves, using hourly data from the RUPE0455, RUPE0738, and RUPE0939 stations. It was shown that the discharge data at station RUPE0738 is biased due to location and ice effects that alter the flow when an ice cover is present (Dupuis 2010, Environnement Illimité INC, 2011); thus, this station was used for ice-free periods only.

## Results

### Saltwater intrusion

**Fig. 5:**
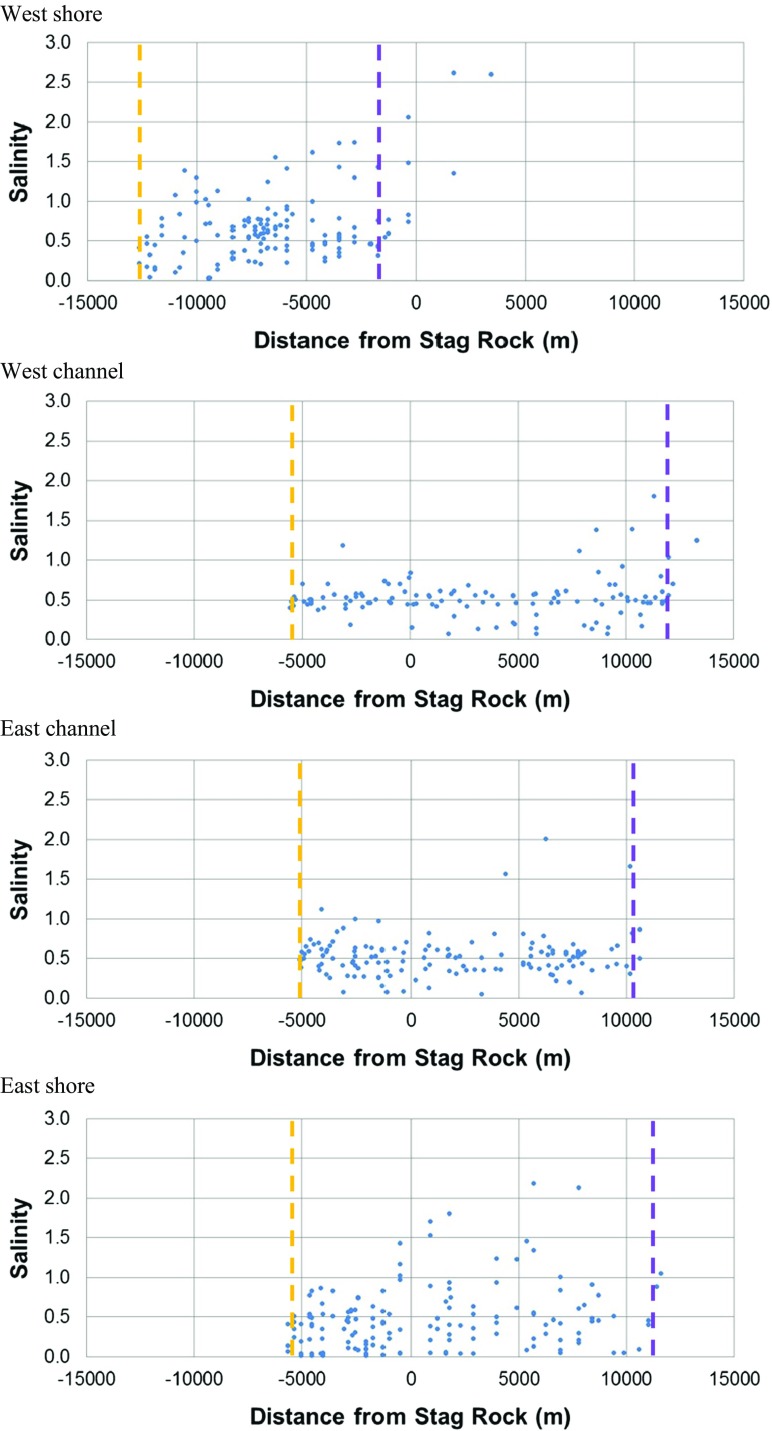
Salinity as a function of distance from Stag Rock (positive in the downstream direction) in open-water conditions during the 2013 monitoring. Yellow dashed line: intrusion of the saline front. Purple dashed line: retreat of the saline front

**Fig. 6:**
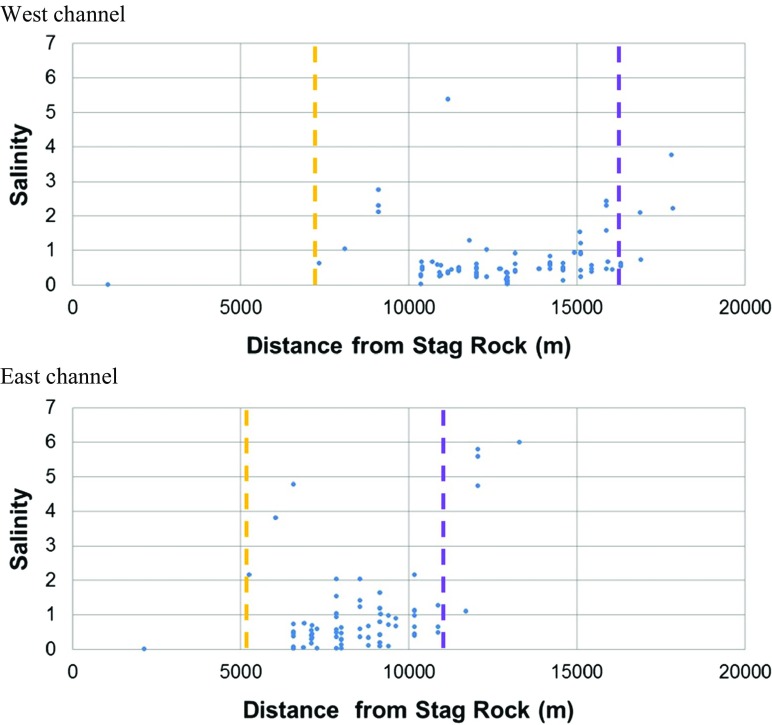
Salinity as a function of distance (positive in the downstream direction) from Stag Rock in ice cover conditions during the 2013 monitoring. Yellow dashed line: intrusion of the saline front. Purple dashed line: retreat of the saline front

Discrete salinity data were used to evaluate the limits of saline intrusion. During the summer of 2013 campaign, the upstream location where salinity reached 0.5 was located approximately 5 to 6 km upstream of Stag Rock in both channels of the Rupert Bay and on the east shore (Fig. 5). On the west shore, the upstream intrusion limit was located more than 10 km upstream of Stag Rock. Under ice cover conditions, the limits of saline intrusion were located downstream compared to open-water conditions. Salinity reached 0.5 approximately 7 km downstream of Stag Rock in the west channel and 5 km in the east channel (Fig. 6).

**Fig. 7:**
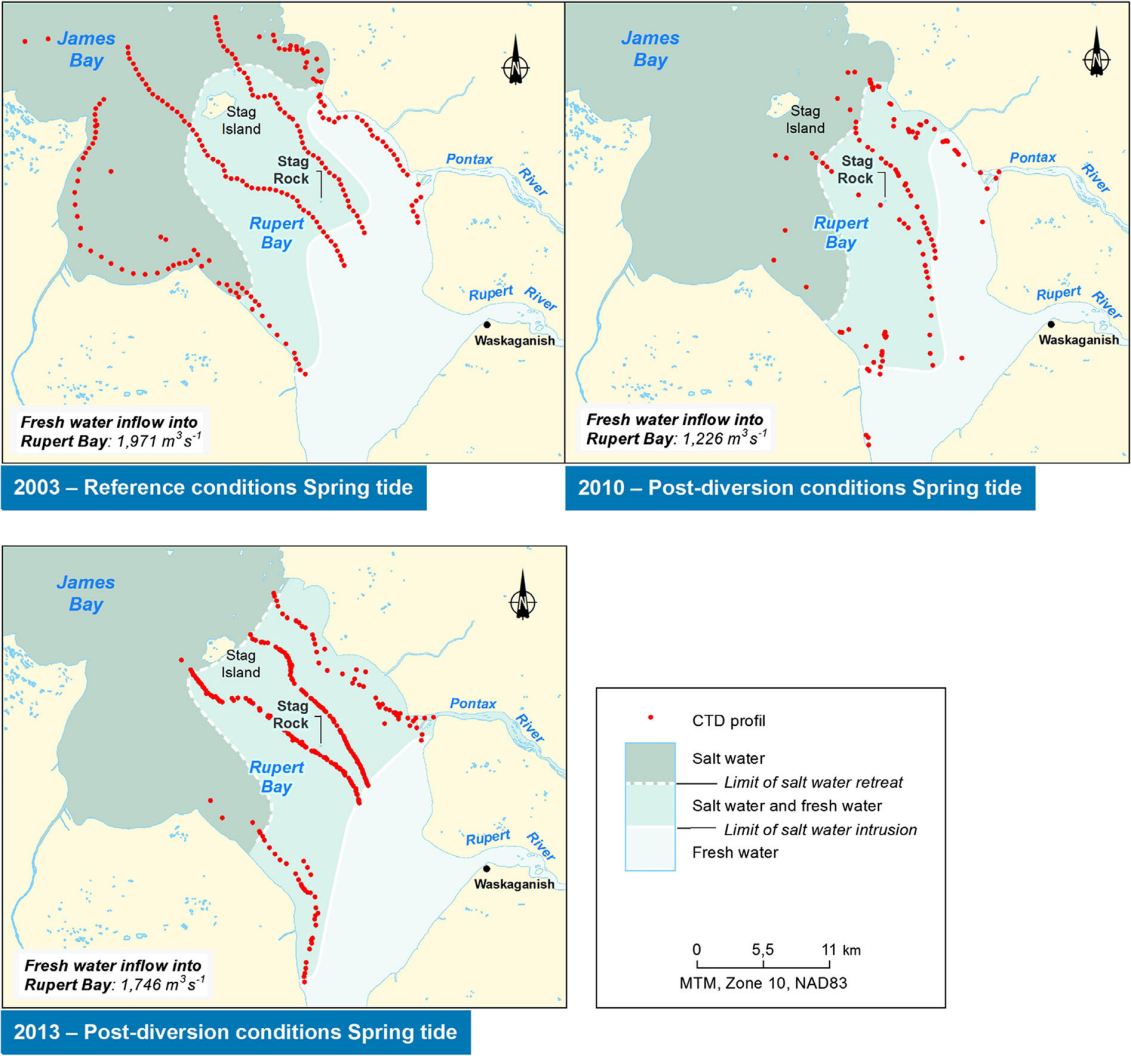
Limits of saltwater intrusion and retreat before and after diversion in open-water conditions

**Fig. 8:**
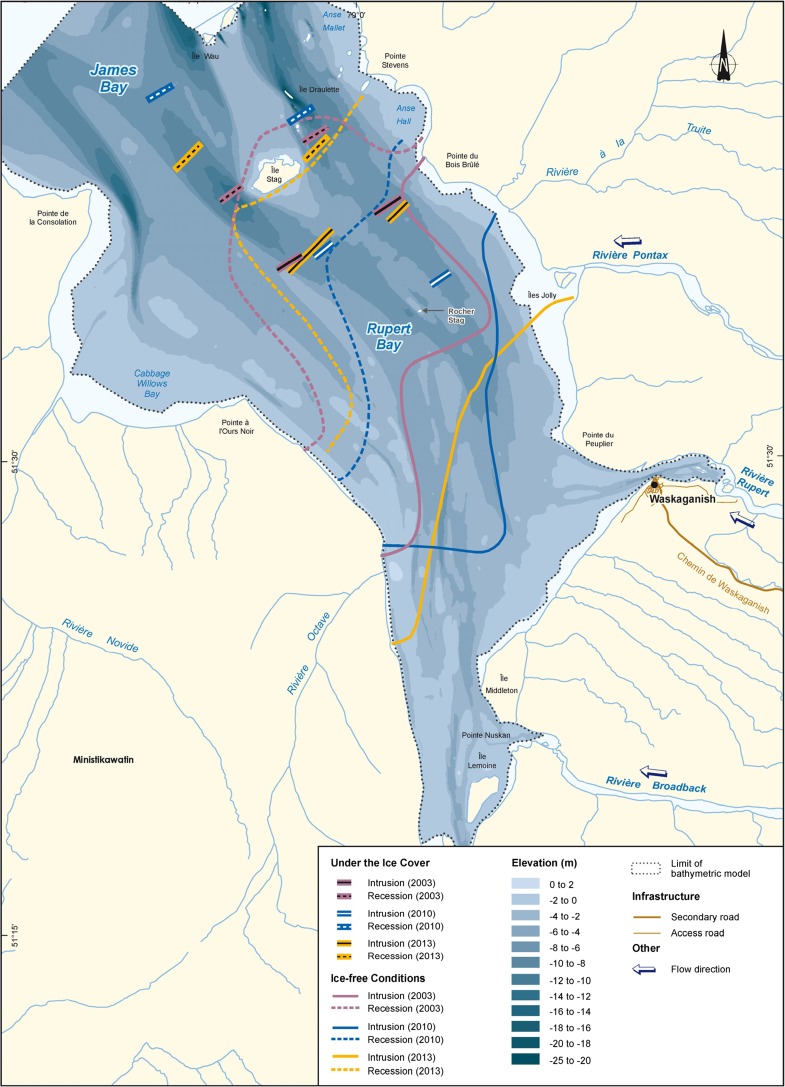
Measured limits of saltwater intrusion in open-water and ice cover conditions

A comparison of pre- and post-diversion conditions shows a saltwater intrusion limit that reaches further upstream of Stag Rock due to a smaller total freshwater inflow (Fig. 7). Since freshwater discharge during monitoring averaged 2971 m^3^ s^−1^ in 2003, 1226 m^3^ s^−1^ in 2010, and 1746 in 2013, the discharge rates during low water of 2010 and 2013 were smaller by 60 and 40% when compared to those of 2003. Before diversion, the limit of saltwater intrusion in the channels was located a few kilometers upstream of Stag Rock, in front of the mouth of the Pontax River. In post-diversion conditions, intrusion moved upstream by 4 to 6 km along the main channels. Onshore, intrusion also appears to have increased, especially on the eastern shore of the bay. In 2003, the upstream boundary of the intrusion on the east bank of the bay was located near the edge of the Bois Brule Point (Fig. 8). In 2013, it reached the mouth of the Pontax River, approximately 8 km upstream. As for the west bank, the limit reached the downstream sector of the Octave River in 2003 and 2010, while in 2013, the intrusion progressed approximately 6 km further upstream toward the mouth of the Rupert Bay.

Under ice cover conditions, the limit of saltwater intrusion has not reached Stag Rock Island for all years of monitoring (2003, 2010, and 2013; Fig. 8), while in open water, it went a few kilometers upstream. This phenomenon is explained by the additional friction caused by the presence of the ice cover which obstructs the water flow and lowers the tidal amplitude. Thus, in winter, saltwater intrusion in Rupert Bay mainly depends on the tide and weather disturbances that significantly affect water levels in the bay. Freshwater inflows in Rupert Bay in 2013 (average of 933 m^3^ s^−1^) are slightly larger than those during previous monitoring years (from 790 to 845 m^3^ s^−1^). Differences in the location of intrusion and retreat of the saline front are mainly related to changes induced by weather fluctuations and the state of the ice cover that prevailed during the limited time span of the field campaign for tracking the movement of the saline front.

### Water levels

**Fig. 9:**
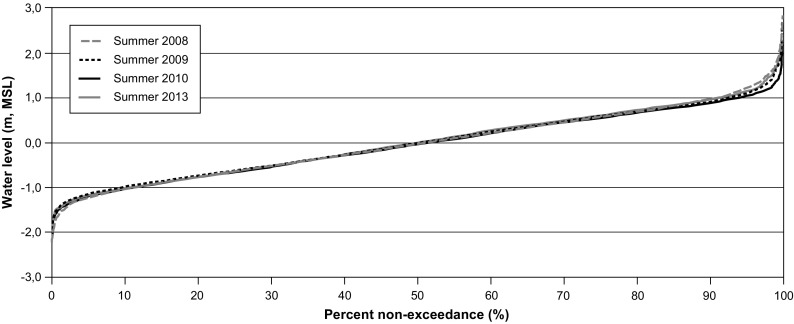
Non-exceedance stage curves at Stag Rock station (RUPE0455) for ice-free conditions during 2008, 2009, 2010, and 2013

Non-exceedance curves show that for all years of observation (Fig. 9), the water surface elevation near Stag Rock oscillates between − 1.0 and 1.0 m about 80% of the time. Despite the smaller freshwater inflow for all the rivers in 2010, non-exceedance curves during 2010 and 2013 show similar responses, with only slight differences for extreme water level events. Weather conditions such as wind velocity and atmospheric pressure are likely responsible for these differences. A comparison between non-exceedance stage curves for pre- and post-diversion conditions shows that there is no significant effect of the diversion on the water levels in the Rupert Bay.

**Fig. 10:**
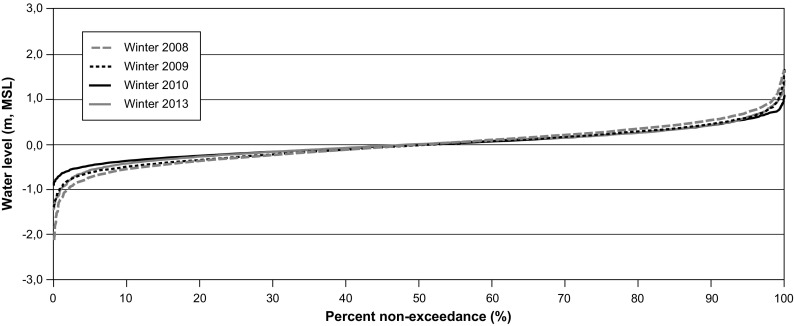
Non-exceedance stage curves at Stag Rock station (RUPE0455) for ice cover conditions during 2008, 2009, 2010, and 2013

Under ice cover conditions, non-exceedance curves show that for all years (Fig. 10), water levels fluctuate between − 0.5 and 0.5 m about 80% of the time. Compared to open-water conditions, the presence of ice cover causes larger interannual variations. Non-exceedance curves for years 2010 and 2013 show discrepancies, especially for extreme values. These differences can be explained by differences in weather conditions and by the variability in the time period needed to develop the ice cover over the entire bay area. For non-exceedance percentages above 10% and below 90%, interannual differences in water levels were small despite a difference of more than 40% of freshwater discharge into Rupert Bay for periods under ice cover between 2008 and 2010 (see Fig. 2). Finally, despite the yearly differences in freshwater inflows, there is no difference for the median water level.

**Fig. 11:**
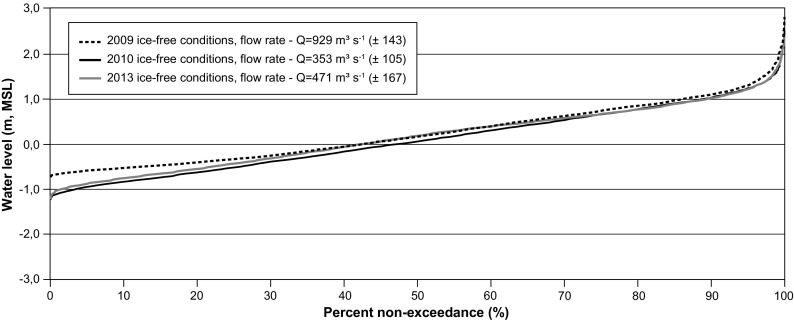
Non-exceedance stage curves at station RUPE0939 for ice-free conditions during 2009, 2010, and 2013

In post-diversion conditions, water levels measured in the estuary of the Rupert River at station RUPE0939 are lower than those measured in pre-diversion conditions (Fig. 11, close to Waskaganish village). The 10-cm decrease in the mean water level is explained by a decrease in the mean river discharge. River-like response at low tide reveals the influence of the diversion on the water levels in the estuary. The decrease reached 30 cm for extreme low water at station RUPE0939 for the time period of analysis.

**Fig. 12:**
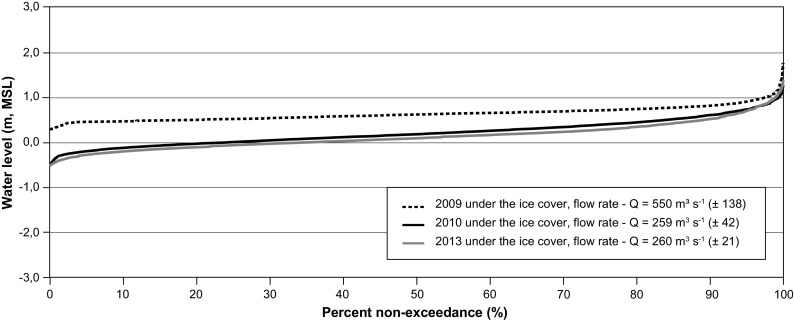
Non-exceedance curves at station RUPE0939 in ice cover conditions during 2009, 2010, and 2013

In winter, the tidal range in the bay and in the estuary of the Rupert River is much smaller, again because of the presence of ice cover. Non-exceedance curves for the years 2009, 2010, and 2013 show that water levels are below 1 m more than 95% of the time (Fig. 12). For 2010 and 2013, curves show a similar behavior; however, water levels in 2013 were slightly lower than those measured in 2010. The most plausible explanation for the fact that the mean water level is larger in 2010 compared to that in 2013 lies in the different conditions of ice cover and different ice characteristic conditions.

## Discussion

### Effects of discharge on saltwater intrusion

**Fig. 13:**
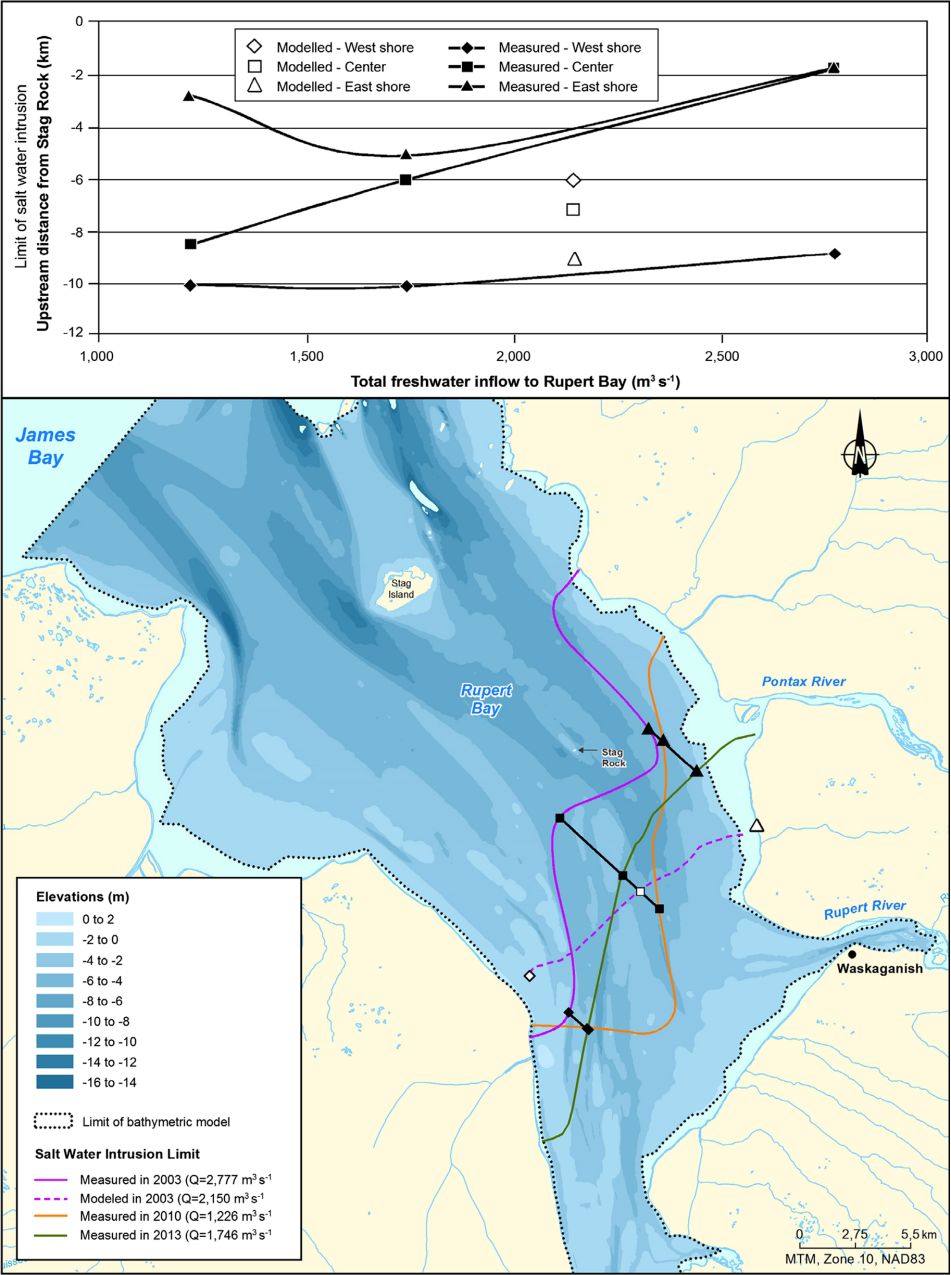
Modeled and measured limits of saltwater intrusion based on discrete measurements in relation with freshwater inflow to Rupert Bay in open-water conditions

During 3 years of saltwater intrusion monitoring in open-water conditions, total river discharge varied by more than a factor of 2 (range from 1226 to 2971 m^3^ s^−1^). The combination of the Rupert diversion with an unusual small runoff in 2010 enabled the study of the influence of total discharge on saltwater intrusion in the Rupert Bay for a wide range of freshwater inflow conditions. Saltwater intrusion along the channels decreases as total river discharge increases. The effect of river discharge on the river plume extent is a well-known phenomenon on the Connecticut River (Garvine 1974, 1975, 1977, 1982), on the Great Whale River (Ingram, 1981), and on the Fraser River (Cordes et al., 1980). It is also discussed on a much larger scale in Prinsenberg (1994) and Déry et al., 2005) and more specifically for diversions, where the effects of multiple river discharges in large bodies of water are studied. In this last paper, natural and anthropogenic impacts on river discharge (such as river diversions) are addressed. Déry et al. suggested that the annual upper-ocean minimum salinity observed on the Newfoundland Shelf can be explained by freshwater pulses composed of meltwater from three successive winter seasons in the river basins draining into the Hudson, James, and Ungava Bays. Moreover, they suggested that a gradual salinization of the upper ocean during summer over the period of 1966–1994 on the inner Newfoundland Shelf is in accord with a decadal trend of a diminishing intensity in the continental meltwater pulses. For the present case and for this range of total river discharge, an increase in river discharge results in a decrease of the saltwater incursion limit distance along the channels (Fig. 13).

Because of tidal flats and shallow waters, saltwater intrusions are episodic near shore, being more subject to weather conditions and wind setup as compared with the saltwater intrusion in the channels. Dupuis (2004), based on a numerical modeling, evaluated that the disruption of normal salinity incursion patterns due to storms fades after a few days. The influence of offshore winds on the along-estuary salinity gradient and residence time was observed by Geyer (1997) on Childs River. In Rupert Bay, offshore winds tend to increase the residence time of saltwater near the banks and increase saltwater intrusion in terms of surface area.

### Effects of ice cover on saltwater intrusion

Under ice cover conditions, our study showed that the river’s freshwater plume extends much more in the downstream direction and covers a larger surface area than in open water (Fig. 8), despite the reduced freshwater discharge during the winter. This observation is consistent with previous studies such as Freeman (1982), Ingram (1981), and Ingram and Larouche (1987a) on Great Whale River as well as Messier et al. (1986, 1989) and Ingram and Larouche (1987b) on La Grande Rivière. The larger extent of the plume is associated with the additional friction associated with the presence of the ice cover which lessens the wetted area and obstructs the incoming saltwater inflow driven by tides. It results in a flatter tidal wave amplitude signal and less energy to push the saltwater front upstream. Indeed, Prinsenberg (1986) found that tidal currents in Hudson Bay are reduced by 20% during the winter as a result of the increased frictional dissipation due to the ice cover.

A power law relationship between surface plume area and river runoff under ice cover is proposed in Ingram and Larouche (1987a) and Messier and Anctil (1996). However, for the cases under study, the mouth of these rivers is located straight across linear open coasts for which the assumption of a semicircular plume area could be realistic (Leblond et al., 1996), regardless of the bathymetry, without any consideration for tidal-induced currents. On the other hand, Rupert Bay’s geometry is such that it could be described as a very wide and shallow channel (mean depth less than 10 m), so that the model is not likely to evaluate surface plume area properly. As discussed earlier, differences in locations and displacement of the salinity intrusion front are mainly related to weather fluctuations and the state of ice cover that prevailed during the limited field campaign time span when the tracking of the movement of the saline front was performed.

### Impact assessment process

For Rupert Bay, the main changes predicted in the project environmental impact assessment were as follows (Hydro-Québec, 2004):

- a decrease in current velocities in the Rupert River upstream of Waskaganish village
- a decrease of 60 to 70 cm of water levels at low tide, within the upper estuary of the Rupert River, upstream of Waskaganish village
- a displacement of the maximum intrusion of saline front of 5 km further upstream within the Rupert Bay
- a slight shift of the freshwater flow corridors from the Nottaway, Rupert, and Broadback Rivers in Rupert Bay toward the northeast
- no noticeable variation in water levels in Rupert Bay.

Changes in velocity and water level upstream of Waskaganish village were predicted based on the expected decrease in water discharge stemming from the diversion of the river 314 km upstream. Although the focus of the current study was the changes in the bay area, the available data on water levels near the river’s mouth are consistent with a 60- to 70-cm decrease (Consortium Waska-Genivar, 2014).

The last three predictions may be evaluated based on the present study.

A comparison between numerical predictions (Fig. 13; Dupuis, 2004) and monitoring results shows that the numerical model over-predicted the displacement of the upstream limit of the saline front by about 3 km along the channels. These observations show the conservative nature of the model which may be explained by a conservative adjustment of the dispersion-diffusion coefficient. Furthermore, the model does not represent the asymmetry observed in the saline front as measured in situ. Asymmetry is characterized by a more pronounced intrusion of the saline front on the west shore and less intrusion on the east shore of the bay. The most likely hypothesis to explain the differences is related to the effects of the Coriolis force which was neglected in the numerical model (Consortium Waska-Genivar, 2014).

The EIA also predicted a displacement of flow corridors of Nottaway, Rupert, and Broadback Rivers in Rupert Bay. The increase in saline intrusion on the west side of the bay may be a manifestation of this displacement of freshwater flow from the Nottaway, Rupert, and Broadback Rivers.

As predicted in the EIA, the comparison of water levels measured at Stag Rock during 2008, 2009, 2010, and 2013 monitoring shows that the partial diversion of the Rupert River has no noticeable effect on water levels in Rupert Bay.

## Conclusion

Rupert Bay is a particular site being a large estuary with very shallow depths which receives a large volume of freshwater inflow from three major rivers on its eastern boundary and where tidal forcing and weather conditions play a major role in water level variations.

In order to improve the knowledge of Rupert Bay hydrodynamics and to understand the impacts of diversion of one of its major tributaries on the saltwater intrusion and the limit of these zones, more than 15 studies, combining oceanographic and hydrological measurements to numerical modeling, were conducted since 1977.

After the diversion, the limit of saltwater intrusion appears to have moved upstream by 4 to 6 km along the main channels. Onshore, intrusion also appears to have increased, especially on the eastern shore of the bay. Under ice cover conditions, the limit of saltwater intrusion has not reached Stag Rock Island for all years of monitoring, while in open water, it went a few kilometers upstream. No noticeable variations in water levels in Rupert Bay were observed after the diversion.

Overall, a good agreement between numerical predictions and monitoring results is observed. Especially, the trends and order of magnitude of the changes in saltwater intrusion are confirmed by salinity and hydraulic measurements in Rupert Bay. Monitoring is also consistent with the changes predicted in the EIA which appear a posteriori conservative.

Monitoring, undertaken in 2010 and 2013 and relying exclusively on empirical data collected both in open-water and ice cover conditions, confirms the predicted trends and order of magnitude of changes anticipated by Rupert Bay EIA. This highlights the good overall performance of the impact assessment and validation process carried out for the Rupert Bay over the years, where the impact of the diversion was first predicted with an acceptable level of precision and was confirmed and refined post-diversion using only empirical measurements during 2 years of operation of the project.

## Footnotes

1 All data used in this study is the property of Hydro-Québec and may be obtained by a request to A. Tremblay.
